# Targeting a transcription factor NF-κB by green tea catechins using *in silico* and *in vitro* studies in pancreatic cancer

**DOI:** 10.3389/fnut.2022.1078642

**Published:** 2023-01-11

**Authors:** Mohd Suhail, Mohd Rehan, Mohammad Tarique, Shams Tabrez, Amjad Husain, Torki A. Zughaibi

**Affiliations:** ^1^King Fahd Medical Research Center, King Abdulaziz University, Jeddah, Saudi Arabia; ^2^Department of Medical Laboratory Sciences, Faculty of Applied Medical Sciences, King Abdulaziz University, Jeddah, Saudi Arabia; ^3^Department of Child Health, School of Medicine, University of Missouri, Columbia, MO, United States; ^4^Innovation and Incubation Centre for Entrepreneurship (IICE), IISER Bhopal, Bhopal, India

**Keywords:** catechin derivatives, epicatechin (EC), epicatechin-3-gallate (ECG), epigallocatechin (EGC), epigallocatechin-3-gallate (EGCG), NF-κB, pancreatic cancer

## Abstract

Pancreatic cancer remains a lethal disease and a major public health problem globally. Nuclear factor-kappa B (NF-κB) has been identified as a therapeutic target in several cancers and plays an important role in inflammatory responses. Many phytochemicals, including catechins, have been reported in the scientific literature with efficient anticancer potential and minimal side effects. This study aims to gain insights into the inhibitory mechanism of catechin derivatives epicatechin (EC), epigallocatechin (EGC), epicatechin gallate (ECG), and epigallocatechin gallate (EGCG) using *in silico* and *in vitro* studies especially considering NF-κB targeting. We explored the binding pose, interacting residues and molecular interactions for catechin derivatives with NF-κB. Docking analysis showed that the catechin derivatives acted as covalent inhibitors with the p65 subunit of NF-κB and interacted with other residues through non-bonding interactions and hydrogen bonds. Further, we validated the effect of EGCG on NF-κB activity in pancreatic cancer cell lines MIAPaCa-2 and SU 86.86. Our *in vitro* data showed EGCG effectively reduced cell growth and proliferation, induced apoptosis, and inhibited NF-κB activity in the studied cell lines. In addition, EGCG repressed the expression of NF-κB target genes including MMP9, MMP2, cMyc, and BCL-2. Thus, targeting NF-κB with EGCG could be a potential therapeutic alternative for pancreatic cancer treatment.

## 1. Introduction

Cancer is still an enigma and a serious public health issue globally. Among the different types of cancer, pancreatic cancer has been one of the most lethal malignant neoplasms for a long time. It is the seventh major cause of cancer-related death worldwide and the 12th most prevalent cancer (1). GLOBOCAN 2020 estimates 496,000 new cancer cases and 466,000 deaths in 2020 (1). In the United States, pancreatic cancer is now the third most common cancer-related cause of death (2). It is reported that overall 5-year survival rate for pancreatic cancer still stands at 10% only, which is significantly very low (3). Its highly aggressive nature and poor survival rate make it a critical global burden (4). The available therapies for this type of cancer have many side effects that demand a novel approach to control its progression. Inflammation is one of the hallmarks of cancer, and nuclear factor-κB (NF-κB) plays a crucial role in inflammatory responses. NF-κB has been established as a therapeutic target in several cancers (5). A study has demonstrated the role of NF-κB as a master regulator of transcription and found its effect in the expression of around five hundred genes (6). NF-κB plays a vital role in regulating the expression of essential regulatory genes such as TNFA, IL6, BCLXL, BCL2, BCLXS, XIAP, VEGF, MMP9/2, Myc, and other target genes. In addition, NF-κB mediates immunity, inflammation, cell proliferation, survival, angiogenesis, and apoptosis (7). The initiation, development, metastasis, and resistance of human cancer are significantly influenced by NF-κB (8, 9). Scientific studies suggested constitutive activation of NF-κB in various cancers, such as breast cancer and prostate cancer, especially in 83% of pancreatic cancer cell lines (9–11). In pancreatic cancer cells, constitutive and induced activation of NF-κB is closely associated with inflammation, cell proliferation, invasion, angiogenesis, anti-apoptosis, and chemotherapeutic resistance (12). The transcription factor NF-κB can be found as a homo or heterodimeric complex (13). Rel-like domain-containing proteins RelA (p65), RelB, p105 (NFB1), (p50) NFB1, Rel, and p52 (NFB2) make up these complexes. Due to the importance of NF-κB, it has been difficult to target this transcription factor in cancer cells (14). However, some previous studies have suggested that there are agents for activation of NF-κB, including carcinogen, tumor promoters, chemotherapeutic agents, and inflammatory cytokines (15). In addition, the NF-κB signaling pathway also is a crucial player in cancer progression and development (16). There are two important pathways; canonical and non-canonical, which regulate heterotrimeric complex of NF-κB (17) comprised of subunits p50, p65, and IκBα in the cytoplasm, where NF-κB resides in the inactive form. With the degradation of IκBα subunit, p50–p65 heterodimer moves to the nucleus, binds the DNA, and activates the target gene. The IκB kinase (IKK)/NFκB) signaling is often altered in human cancers and is considered an important signaling pathway for cancer progression. Different mouse models of cancer wherein IKK/NFκB activation has been obstructed genetically have shown that NF-κB is a key promoter of inflammation-associated cancers (18–20). Some studies suggested that the chemical inhibitors of human NF-κB could successfully minimize the onset and progression of multiple cancers.

**Figure 1:**
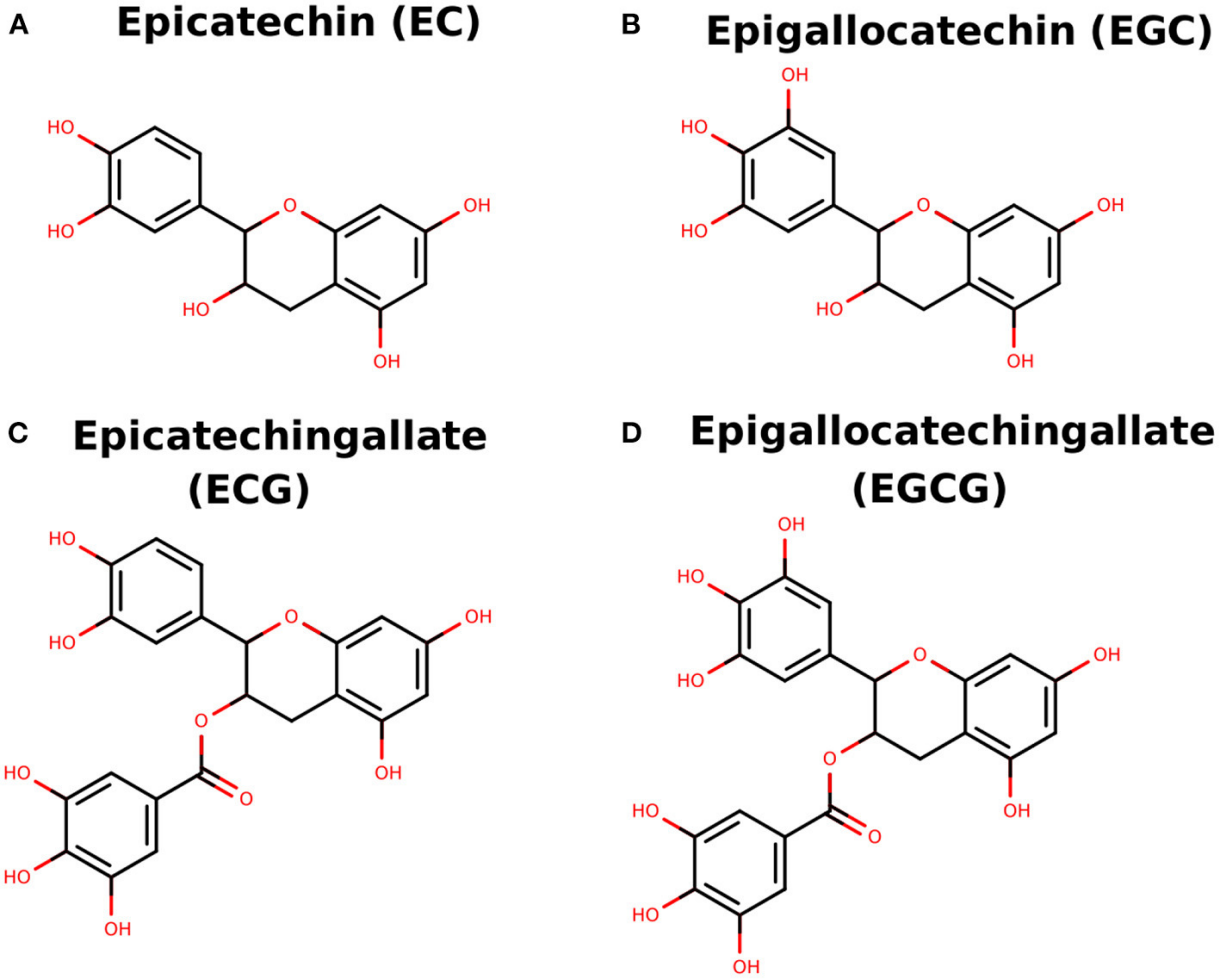
**(A–D)** Two-dimensional sketch of EC, EGC, ECG, and EGCG.

Several phytochemicals have been reported with efficient anticancer potential and minimal or no side effects. The natural compounds catechin derivatives, generally obtained from green tea, are well-known for their anticancer activities (21). Green tea (GT) is a popular refreshing drink consumed by people across the world. The plant *Camellia sinensis*, which is majorly found in Asian countries, is the main GT source (22). Several shreds of evidence have shown that green tea has antimicrobial, anti-carcinogenic, antioxidant, and anti-inflammatory properties and its potential benefits in neurologic disorders, diabetes, oral health, obesity, and cardiovascular disease are well-known (23). The anti-carcinogenic properties of green tea include controlling cell proliferation, cell death in tumor cells, and vascular angiogenesis in solid tumors. Recent studies have shown that catechins found as a natural compound in green teas have an inhibitory effect against NF-κB. The principal four vital components in GT as catechins are 1) EC, (-)-epicatechin 2) EGC, (-)-epigallocatechin 3) ECG, (-)-epicatechin-3-gallate and 4) EGCG, (-)-epigallocatechin-3-gallate (Figure 1). Among these catechins, EGCG is the richest compound in green tea (24). Various computational methods have increasingly been used for exploring binding pose, interacting residues, and novel drug designing (24–30). In the current study, we analyzed the detailed analysis of binding pose and molecular interactions of catechin derivatives to NF-κB using computational methods. Further, EGCG was tested and validated in an experimental laboratory.

## 2. Materials and methods

### 2.1. Data retrieval

The 3-D coordinates of NF-κB p65 subunit were retrieved from PDB with PDB Id, 1NFI, and the 3-D coordinates of catechin derivatives EC, ECG, EGC, and EGCG were retrieved from PubChem with CIDs 72276, 107905, 72277, and 65064, respectively.

### 2.2. Covalent docking

For covalent docking, the covalent bonds were formed between the S-atom of Cys-38 and the reported atom of EGCG and other catechins (31). The conformation of the Cys-catechin adduct was relaxed by making it flexible for rotation through various bonds while docking a small molecule, water. The relaxed conformation of Cys-catechin adduct so obtained was reported as the final covalent docking result. Flexible docking was done with Autodock Vina (32), and protein, ligand, and grid boxes were prepared by Autodock Tools (33). Pymol (34) and Ligplot+ (35) were used for protein-ligand complex analyses and generating final illustrations.

### 2.3. Chemical and reagents

EGCG (≥95%) of culture grade was purchased from Sigma-Aldrich (St. Louis, MO, USA). The following items were purchased from Mediatech, Inc. (Herndon, VA, USA), including trypsin, fetal bovine serum (FBS), McCoy's 5A, and Leibovitz's L-15 media, phosphate-buffered saline (PBS), penicillin/streptomycin solution, propidium iodide (PI), and RNase. NF-κB p65 transcription factor assay kit (ab133112) was procured from Abcam, USA. A cellTiter 96 aqueous one solution kit was purchased from Promega (Madison, WI, USA) for cell proliferation assay and 3-[4,5-dimethylthiazole-2-yl]-2,5-diphenyltetrazolium bromide (MTT) was purchased from Thermofisher, USA.

### 2.4. Cell culture

Human pancreatic cell lines, MIAPaCa-2 (ATCC^®^CRM-CRL-1420) and SU.86.86 (ATCC^®^ CRL-1837), were obtained from ATCC and were cultured according to the recommended conditions. The cells were grown at 37°C in a humidified environment with 5% CO2 in DMEM medium supplemented with 5% FBS and 50 IU penicillin/streptomycin. When the cells were confluent, healthy, and free of contamination, cell culture media was removed, and cells were washed with PBS to remove any remaining serum that would have rendered the trypsin inactive. One ml trypsin was added to a 25 mL flask to break the cell-cell and cell-substrate links. To inactivate the trypsin, 5 ml fresh culture medium with serum was added to the cell suspension, and a single cell suspension was prepared after pipetting this suspension. The cell density accuracy was confirmed by counting the cell suspension. In the end, an aliquot of cell culture medium was prepared into a 25 mL new flask, and the medium was changed further as per the requirement to get subsequent culture.

### 2.5. Cell proliferation analysis

Human pancreatic cancer cell lines MIAPaCa-2 and SU.86.86 were plated in 24 well-culture plates for the cell proliferation analysis. The complete DMEM medium supplemented with 100 U/mL penicillin and 100 U/mL streptomycin was used to grow the cells at 37°C in a humid chamber containing 5% CO2. The final 1% EGCG was prepared in DMSO and stored at −20°C before use. Control cells were treated with only 1% DMSO without EGCG. Both cell lines were separately seeded in a 96-well microtiter plate (~10,000 cells/well). To determine the cytotoxic effect of EGCG, an MTT assay was performed by treating cells with different concentrations (10–100 μM) of EGCG for 24 h. After 24 h of incubation, 20 uL of MTT stock solution was added in each well, and further incubation was done for 3 h. To solubilize the formazan violet crystals, medium was decanted, 500 μl DMSO was added in each cell, and absorbance was measured by ELISA reader at 540 nm (Molecular Devices, Sunnyvale, CA). The percentage cell viability was determined at different concentrations of EGCG (0, 10, 20, 40, 60, 80, and 100 μM) by scheming the graph of cell viability and concentration of the EGCG in both MIAPaCa-2 and SU.86.86 cells. Since 1% DMSO did not affect the proliferation of the two cell lines, results were expressed as a percent of control.

### 2.6. NF-κB activity assay

ELISA-based transcription factor assay kit (ab133112, Abcam) was used to determine the EGCG effect on p65-NF-κB activity in human pancreatic cell lines (MIAPaCa-2 and SU.86.86). The nuclear extract was extracted by lysing the treated cells with EGCG (10–100μM for 24 h) using hypotonic HEPES lysis buffer (pH 7.4) provided with a nuclear extraction kit (ab113474, Abcam). As recommended by the manufacturer, the supernatant was taken and utilized to measure intracellular p65-NF-κB by ELISA. In brief, the p65-NF-κB response element with a specific double-stranded DNA (dsDNA) sequence was immobilized onto the bottom of wells in a 96-well plate. The p65-NF-κB contained in a nuclear extract bind specifically to the p65-NF-κB response elements. The p65-NF-κB was detected by adding a specific primary antibody directed against p65-NF-κB. A secondary antibody that was HRP-conjugated was added, and a spectrophotometer was used to measure the absorbance at 492 nm.

### 2.7. RNA extraction and real time PCR

Total RNA was extracted from pancreatic cancer cells after treatment with various concentrations (0, 20, 60, 100 μM) of EGCG for 24 h, using TRIzol reagent (Invitrogen, Carlsbad, CA, USA), according to the manufacturer's instruction. A UV spectrophotometer was used to measure the quantity and quality of the RNA. All the primes were designed through ww.ncbi.nlm.nih.gov/gene. The cDNA was synthesized using the 1 μg total RNA through cDNA Synthesis Kits (Thermo Scientific, USA) following the manufacturer's instructions. The synthesized cDNA was stored at −20°C for further use. Triplicate samples of cDNA from co-cultured from each group was amplified in 384 well-plates containing primers for the genes of interest, MMP2, MMP9, Myc, BCL-2, and housekeeping gene GAPDH in Roche, LightCycler^®^ 480 System. Data were analyzed as threshold cycle values were normalized and expressed as DCt: mean Ct of the gene of interest – mean Ct.

### 2.8. Apoptosis analysis

MIAPaCa-2 and SU.86.86 cell lines were treated with various doses (0, 20, 60, and 100 μM) of EGCG for 24 h at 37°C to perform cell apoptosis and cell cycle analysis using Annexin V-FITC Propidium Iodide (PI) staining kit (ab14085, Abcam). The control cells were treated with media only. After harvesting the culture, 1 × 105 cells were washed with 5 ml of PBS and trypsinized by adding 3 ml of trypsin at 37°C for 5–10 min. Subsequently, harvested cells were double stained with an Annexin V-FITC/PI. The flow cytometry BD FACS Canto analyzed 10,000 events for each sample, and data were analyzed using FCS express 7 software.

### 2.9. Statistical analysis

Data obtained from various culture experiments were analyzed by one-way ANOVA test for group analysis using Graph Pad Prism version 5 (Graph Pad software, Inc., San Diego, CA). Results are expressed as mean ± SD with at least two independent experiments performed in triplicate. *P* < 0.05 was considered as statistically significant.

## 3. Result and discussion

### 3.1. Covalent docking of catechin derivatives to NF-κB p65 subunit

It is reported that EGCG forms a covalent bond with Cys38 residue of NF-κB p65 subunit and acts as a covalent inhibitor of this protein (31). However, the structural details of binding conformation, interacting residues and molecular interactions have not been explored yet. We attempted to provide the structural insights into covalent binding of catechin derivatives to NF-κB p65 subunit. Notably, there is no cavity containing residue Cys-38, where catechins can go and bind normally involving non-bonded and hydrogen bonding interactions. Therefore, we proceeded for the only option of covalent docking of catechins to NF-κB p65 subunit following the earlier reported study (31). All the four catechins formed an adduct with Cys-38 residue, which hangs on the surface of the protein.

**Figure 2:**
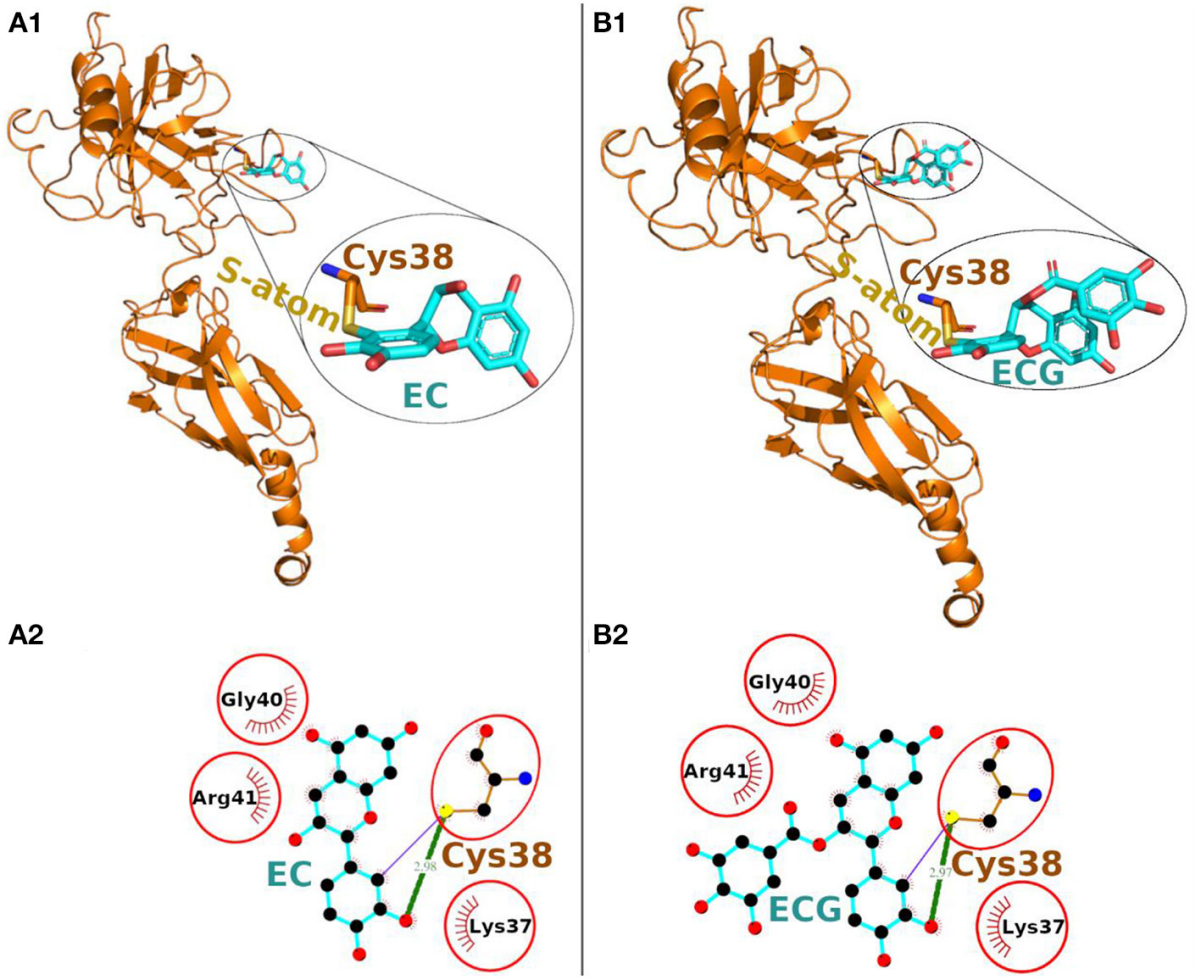
Covalent docking of EC **(A)** and ECG **(B)** to NF-κB p65. **(A1, B1)** The protein is shown in ribbon representation colored orange with Cys adducts. In inset, the Cys adduct is zoomed and shown in sticks representation with Cys-38 backbone colored orange, S-atom colored yellow and EC and ECG backbones in cyan. The O-atoms and N-atoms are colored in red and blue colors, respectively. **(A2, B2)** Ligand-protein interaction plots. The EC and ECG with Cys-38 are shown with backbone color cyan and orange, respectively with balls as atoms. The color of the balls decides atom types as the black color for C-atoms, the blue for N-atoms, red for O-atoms and yellow for S-atoms, respectively. The covalent bond with S-atom of Cys-38 is shown as purple line and the hydrogen bond as green line with bond length in Å. The residues forming non-bonded contacts are shown as red comb like structure. The residues found common to the interacting residues of EC and ECG are encircled.

The Epicatechin (EC) formed a covalent bond with sulfhydryl S-atom of Cys-38 and the final conformation of Cys-EC adduct is shown in Figure 2A1. In addition, the Cys-38 also formed a hydrogen bond and 12 non-bonded contacts (Figure 2A2, Table 1). Other residues in the neighborhood of Cys-38 also interacted with EC. Four interacting residues, Lys-37, Cys-38, Gly-40, and Arg-41, contributed to one covalent bond, one hydrogen bond and 21 non-bonded contacts.

**Table 1:** Interacting residues of NF-κB p65 for EC and ECG.

**Catechins**	**Interacting residues**	**Covalent bonds**	**Hydrogen bonds**	**Non-bonded contacts**
EC	Lys-37			1
	Cys-38	1	1	12
	Gly-40			7
	Arg-41			1
ECG	Lys-37			1
	Cys-38	1	1	13
	Gly-40			8
	Arg-41			1

The number of molecular interactions including covalent bonds, hydrogen bonds, and non-bonded contacts are provided for each interacting residue.

Another catechin ECG, galloyl derivative of EC, was also subjected to covalent docking. The final conformation of the Cys-ECG adducts was similar to that of EC with same set of interacting residues as shown in zoomed insets of Figures 2A1, B1, respectively. The Cys-38 forms a covalent bond, a hydrogen bond and 13 non-bonded contacts with ECG. The four interacting residues Lys-37, Cys-38, Gly-40, and Arg-41 (similar to that of EC) contributing together to one covalent bond, one hydrogen bond and 23 non-bonded contacts (Figure 2B2, Table 1).

**Figure 3:**
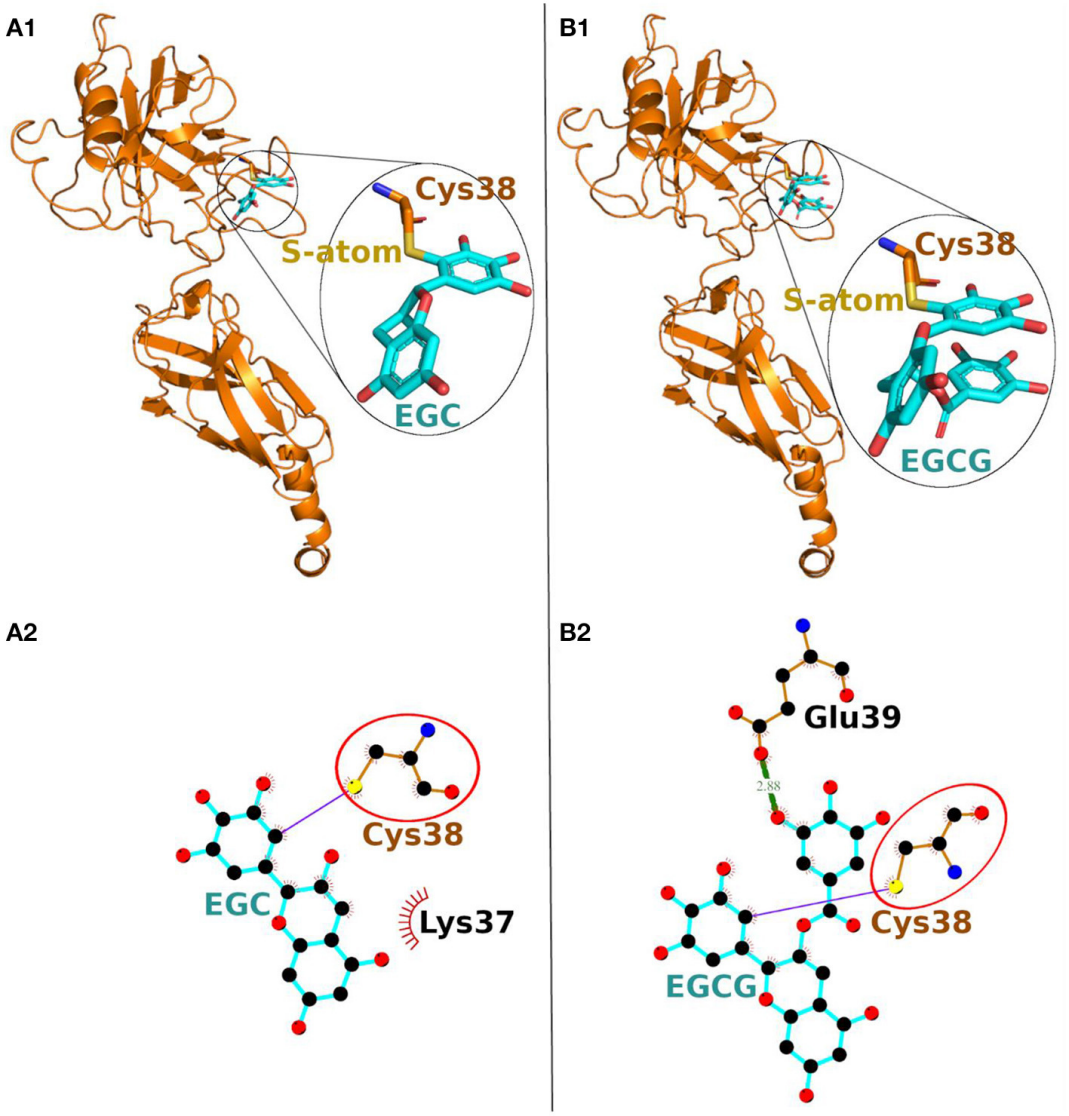
Covalent docking of EGC **(A)** and EGCG **(B)** to NF-κB p65. **(A1, B1)** The protein is shown in ribbon representation colored orange with Cys adducts. In inset, the Cys adduct is zoomed and shown in sticks representation with Cys-38 backbone colored orange, S-atom colored yellow and EGC and EGCG backbones in cyan. The O-atoms and N-atoms are colored in red and blue, respectively. **(A2, B2)** Ligand-protein interaction plots. The EGC and EGCG with Cys-38 are shown with backbone color cyan and orange, respectively with balls as atoms. The color of the balls decides atom types as the black color for C-atoms, the blue for N-atoms, red for O-atoms and yellow for S-atoms, respectively. The covalent bond with S-atom of Cys-38 is shown as purple line and the hydrogen bond as green line with bond length in Å. The residues forming non-bonded contacts are shown as red comb like structure. The common interacting residue, Cys-38 is encircled.

On the other hand, in covalent docking of catechin EGC, the Cys-38 formed a covalent bond and 11 non-bonded contacts. Another residue Lys-37, the previous residue to Cys-38 in protein chain, also interacted through two non-bonded contacts (Figure 3A1, Table 2).

**Table 2:** Interacting residues of NF-κB p65 for EGC and EGCG.

**Catechins**	**Interacting residues**	**Covalent bonds**	**Hydrogen bonds**	**Non-bonded contacts**
EGC	Lys-37			2
	Cys-38	1		11
EGCG	Cys-38	1		16
	Glu-39		1	4

The number of molecular interactions including covalent bonds, hydrogen bonds, and non-bonded contacts are provided for each interacting residue.

Finally, the covalent docking of EGCG showed that Cys-38 formed a covalent bond and 16 non-bonded contacts (Table 2). Another interacting residue Glu-39, the next sequential residue of Cys-38, contributed to one hydrogen bond and four non-bonded contacts. The comparable binding conformation of Cys adduct of EGC and EGCG are shown in zoomed insets of Figures 3A1, B1, respectively. Thus, all the catechin derivatives reacted with Cys-38 of NF-κB p65 subunit and act as covalent inhibitors of this protein.

Although, our *in-silico* study showed covalent bonding with Cys-38 of NF-κB p65 by all four catechin derivatives but keeping in mind the abundance (~50–80%) of EGCG in green tea [15], and presence of highest number of hydroxyl groups resulting high antioxidant activity, we stick with EGCG in our future *in-vitro* experiments.

### 3.2. *In vitro* analysis of EGCG on pancreatic cancer cell lines

Due to the high prevalence of pancreatic cancer globally and its link with NF-κB hyperactivity (12, 36), we have used pancreatic cancer cell lines to evaluate EGCG for anticancer activity.

### 3.3. EGCG reduces pancreatic cancer cell growth and proliferation

**Figure 4:**
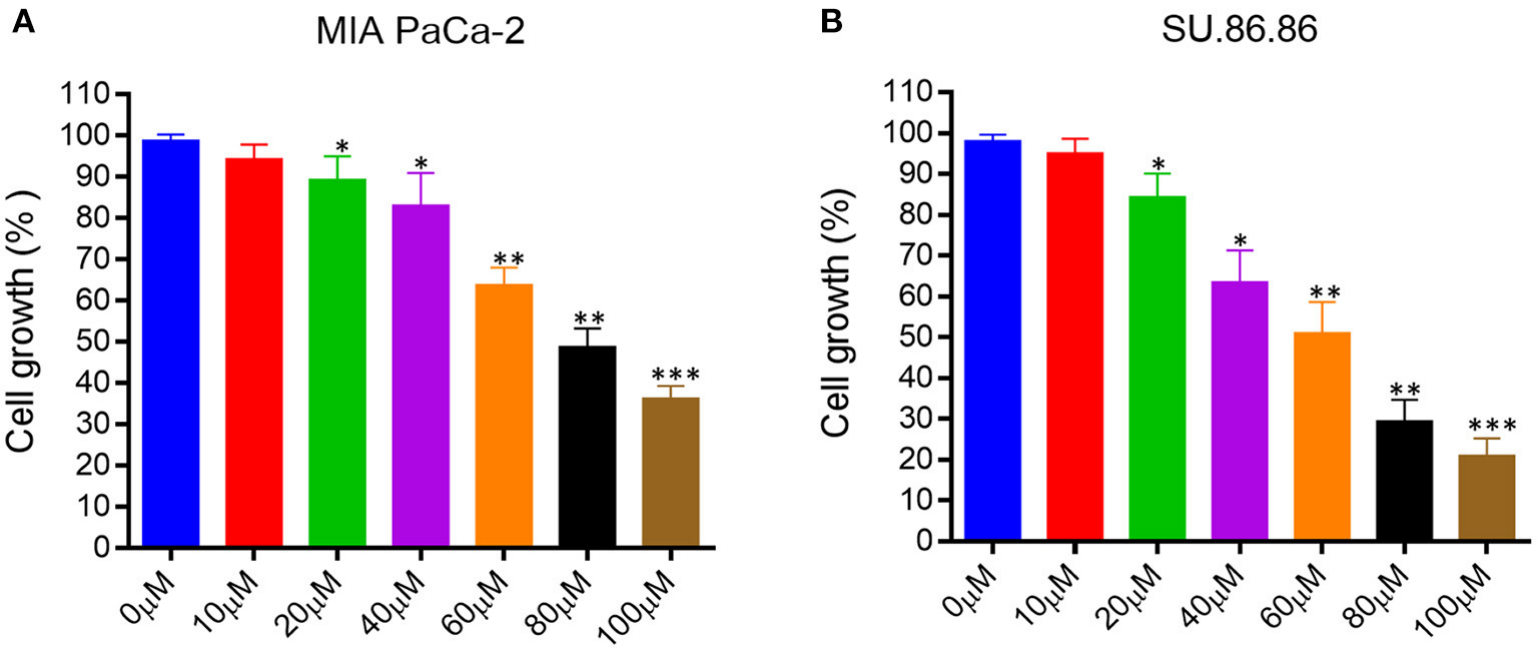
Epigallocatechin gallate (EGCG) inhibits pancreatic cancer cell growth *in vitro*. Human pancreatic cancer cell lines were treated with various concentrations of EGCG (0–100 μM), and cell growth was determined in MIA PaCa-2 **(A)**, and SU- 86.86 **(B)** cells after treatment with increasing EGCG concentrations for 24 h. Cell growth was found to be inhibited in a concentration dependent manner. Results are expressed as a percentage of control and mean ± SD (*n* = 4). *p < 0.05, **p < 0.01, ***p < 0.01 vs. control.

We evaluated the effect of EGCG on proliferation and cell growth in human pancreatic cancer cell lines (MIAPaCa-2 and SU.86.86). For this purpose, we treated MIAPaCa-2 and SU.86.86 with increasing concentrations of EGCG (10–100 μM) for 24 hrs. A significant reduction in the viability and proliferation of MIAPaCa-2 and SU.86.86 cells was observed with increased EGCG concentration (Figure 4). The IC50 values of EGCG against the used cell lines were calculated from the plot of percentage cell viability vs. EGCG concentration. The IC50 of EGCG for MIAPaCa-2 and SU.86.86 were found to be 73 and 59 μM, respectively.

EGCG inhibited the growth of human pancreatic cancer MIAPaCa-2 and SU.86.86 cells in culture in a concentration-dependent manner. These results collectively indicate that EGCG may possess the antiproliferative activity and inhibit the MIAPaCa-2 and SU.86.86 cell proliferation.

### 3.4. EGCG inhibited NF-κB activity in pancreatic cancer cell

Next, we examined the effect of EGCG on NF-κB activity in the studied cell lines. After activation NF-κB pathway, its p65 (Rel A) is translocated into the nucleus, so we looked presence of p65 (Rel A) subunit for NF-κB activity after treating the pancreatic cancer cell lines with EGCG in various concentrations (0, 20, 60, 100 μM). NF-κB is a multidimensional transcription factor, and it regulates the activities of multiple genes that play an essential role in innate immunity and inflammation (37). Therefore, we wanted to examine the impact of EGCG in the modulation of phosphorylated (p65)-nuclear factor-κB (p65-NF-κB) expression in human pancreatic cancer cells.

**Figure 5:**
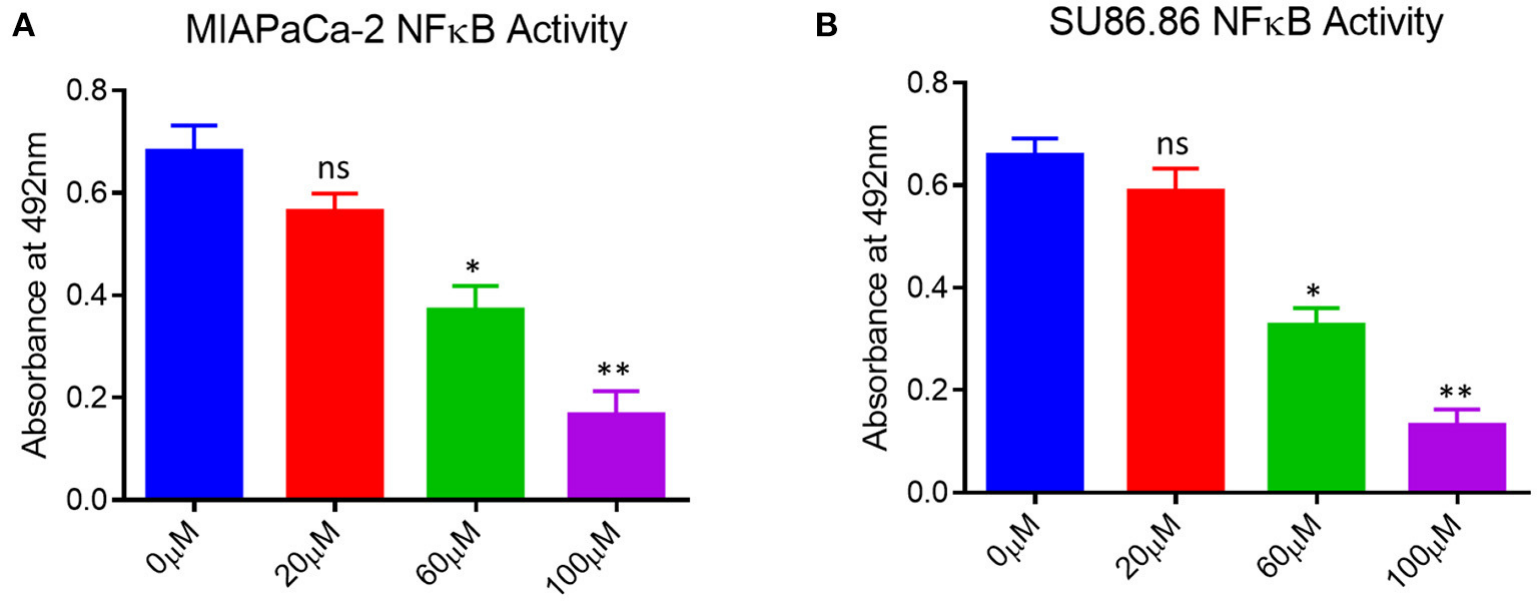
NF-κB activity in untreated (0 μM) and treated **(A)** MIAPaCa-2 **(B)** SU 86.86 with various concentrations of EGCG (20, 60, and 100 μM) cells. MIAPaCa-2 and SU 86.86 cells were seeded at a density of 10^5^/well in 24-well plates and induced with EGCG (20, 60, and 100 μM) for 24 h. Levels of p65-NF-κB activity were up-regulated in untreated human pancreatic cancer cells, and EGCG treatment reduced the p65-NF-κB activity in both pancreatic cell lines at 60 and 100 μM. Results are expressed as a percentage of control and mean ± SD (*n* = 4). ^*^ *p* < 0.05, ^**^ *p* < 0.01, vs. untreated cells.

As shown in Figure 5, the up-regulation of p65-NF-κB in the untreated human pancreatic cancer cell lines and EGCG treatment significantly reduces the p65-NF-κB activity in both the cancer cell lines as compared to the untreated cells. The activation of downstream signaling leads to the proliferation and progression of the cancer cells, indicating the role of p65-NF-κB expressed in the nucleus. These results showed that p65-NF-κB levels were significantly repressed when MIAPaCa-2 and SU.86.86 cells were treated with EGCG specifically at 60 and 100 μM. Thus, in EGCG treated MIAPaCa-2 and SU.86.86 cells, the expression of p65-NF-κB was attenuated and the levels almost toward baseline level, suggesting that EGCG reduces the MIAPaCa-2 and SU.86.86 cells proliferation by inhibiting p65-NF-κB activity in MIAPaCa-2 and SU.86.86 cells.

### 3.5. EGCG induced apoptosis in pancreatic cancer cell

**Figure 6:**
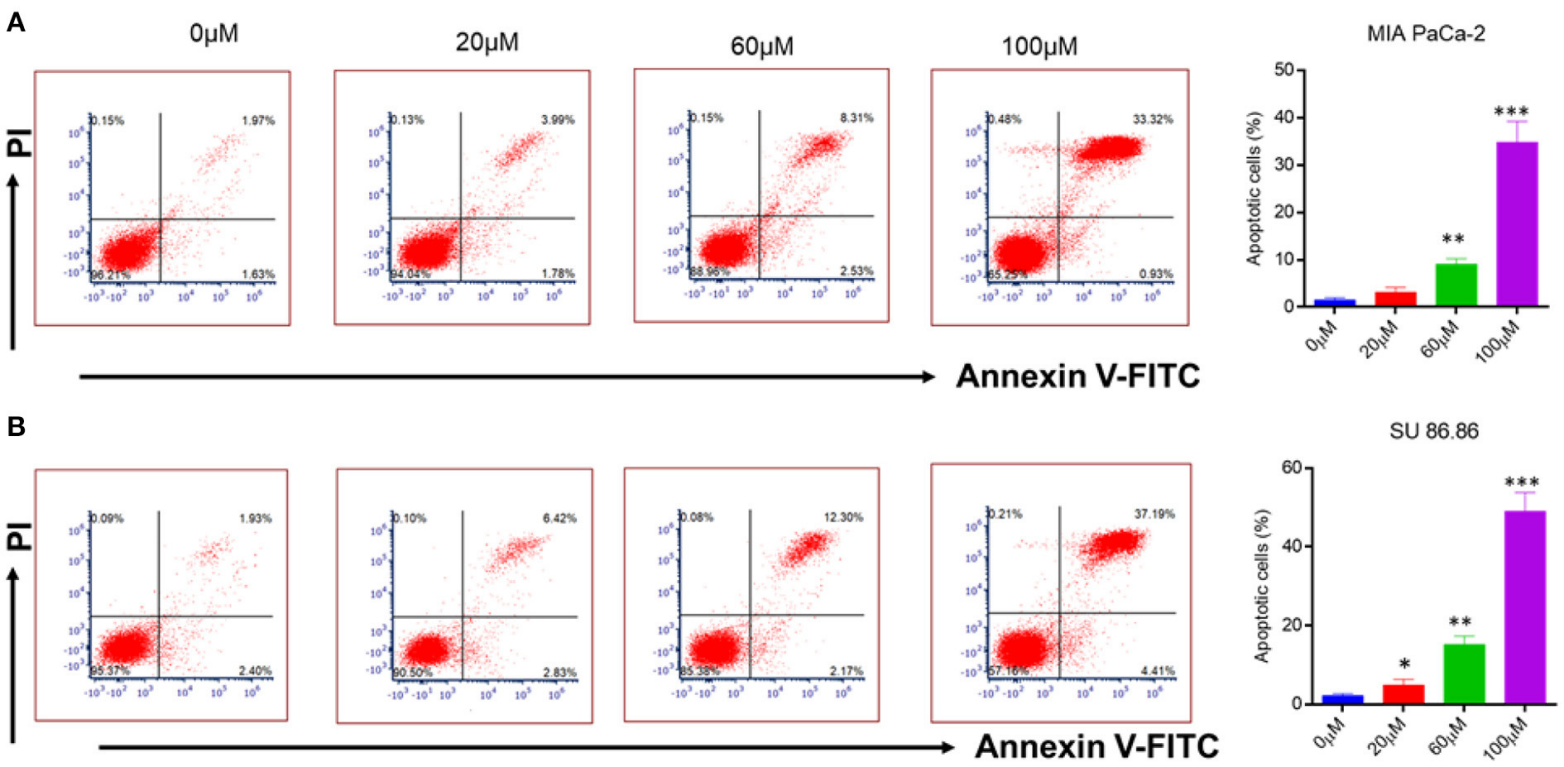
Flow cytometry analysis of annexin V-FITC/PI staining **(A)** MIAPaCa-2 **(B)** SU 86.86 cell lines were cultured with increasing concentration (0–100 μM) of EGCG for 24 h. Annexin V-FITC/PI staining showed an increasing percentage of apoptotic cells with an increasing concentration of EGCG due to cell cycle inhibition. Bar graphs indicating the percentage of apoptotic cells as the concentration of EGCG increases (0–100 μM). The data represent the mean ± SD (*n* = 4) values of triplicate determinations. *p < 0.05; **p < 0.01; ***p < 0.001 compared with the control. Data were analyzed using Flowjo software. ANOVA test was applied in the statistical analysis for group analysis.

To check the anti-cancerous activity of EGCG, human pancreatic cancer cells (MIAPaCa-2 and SU 86.86) were treated with increasing concentrations of EGCG (0–100 μM). The extent of apoptotic cells with the increasing concentration of EGCG is shown in Figure 6.

It is well-known that cancerous cells have the ability to evade apoptosis (38). Therefore, to see whether the suppression of NF-κB by EGCG has any effect on apoptosis, human pancreatic cancer cell lines (MIAPaCa-2 and SU 86.86) were incubated with the increasing concentration of EGCG for 24 h (Figure 6A). In the case of MIAPaCa-2 cell line, upon increasing the concentration of EGCG (20, 60, and 100 μM), a significant increase in the percentage of apoptotic cells (3.13, 9.07, and 34.85%, respectively) was observed.

However, in SU 86.86 cells, a slightly more increase in the number of apoptotic cells (4.92, 15.17, and 49.05%), was observed at 20, 60, and 100 μM concentrations of EGCG (Figure 6B). It suggests that EGCG induces more apoptosis in SU 86.86 cells than MIAPaCa-2 cells. These results suggest that EGCG has anti-cancerous activity on human pancreatic cells and induces significant apoptosis in these cancerous cell lines.

### 3.6. EGCG inhibits NF-κB downstream signaling molecules in pancreatic cancer cell

**Figure 7:**
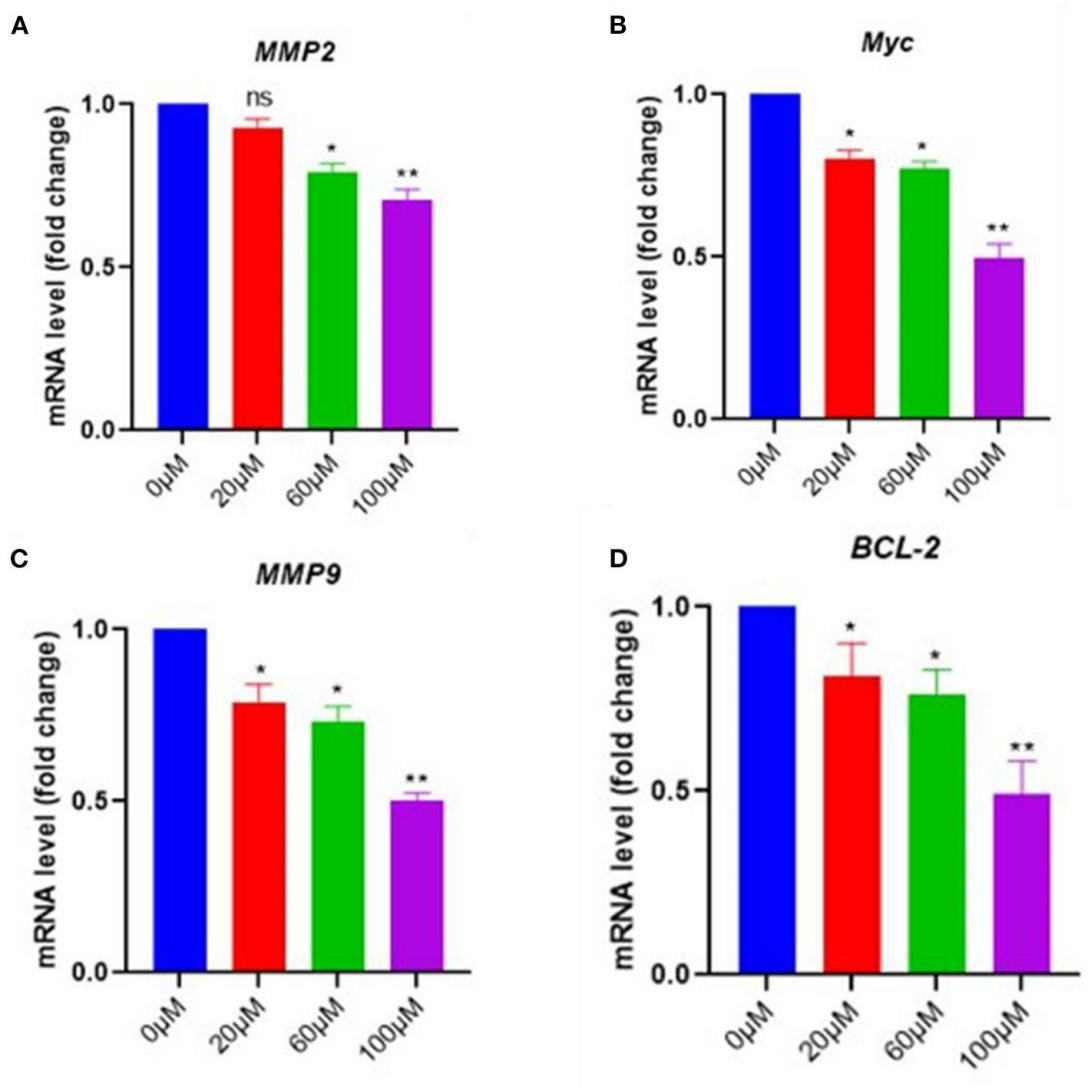
In pancreatic cancer cells, EGCG reduces the RNA expression of NF-κB downstream signaling genes. Real-time PCR (qPCR) results revealed that EGCG administration reduces the expression of NF-κB target genes (e.g., MMP9, cMyc, MMP2, and BCL-2) in pancreatic cancer cells treated with various doses (0–100 μM) for 48 hrs and cells were harvested, and RT-PCR was done for NF-κB target genes **(A–D)** (*n* = 3; ^*^ *p* ≤ 0.05, ^**^ *p* ≤ 0.01). Statistical analysis was done using the ANOVA test for group analysis.

To further confirm the effects of EGCG on NF-kB inhibition, the mRNA level of NF-κB essential targets genes MMP9, MMP2, cMyc, and BCL-2, was determined using quantitative PCR (qPCR). The pancreatic cancer cell line was treated with 0–100 μM of EGCG for 48 h, and mRNA levels of MMP9, MMP2, cMyc, and BCL-2 were determined by RT-PCR. We observed a decrease in the mRNA level of NF-κB target genes after treatment with EGCG, which indicated that EGCG inhibits the NF-κB activity; thus, downstream target gene inhibition was observed. Furthermore, the qPCR results correlated well with the results obtained from the NF-κB activity assay and apoptosis assay, suggesting that EGCG inhibits NF-κB activity. Again, NF-κB inhibition by EGCG treatment represses the expression of MMP9/2 and other NF-κB target genes (Figure 7).

Natural products have been widely used to lessen the side effects of chemotherapy and cancer drugs in cancer treatment (39, 40). Since time, attention has been given to the dysregulation of NF-κB in various cancers (10). Dysregulation of NF-κB affects the regulation activity of several oncogenes, thus leading to tumor progression, inhibition of apoptosis, and cell proliferation (10). An abysmal prognosis and metastatic disease are common pancreatic cancer diagnoses. Since chemotherapeutics currently available offer little help for pancreatic cancer patients, new therapeutic strategies are needed, including a combination of drugs and natural compounds like EGCG with minimal side effects.

Our study evaluated the effect of EGCG on the NF-κB activity in human pancreatic cancer cells. The findings revealed that the NF-κB activity in both human pancreatic cancer cells significantly decreased as EGCG concentration was increased. These results agree with our docking studies. To further evaluate the possibility of EGCG as a treatment to prevent pancreatic cancer, the effect of EGCG on cell cytotoxicity, cell proliferation, cell cycle, and apoptosis was tested in relevant cancer cell lines. It is widely known that NF-κB contributes to the development of several cancers (41–44). However, it was shown that EGCG is particularly more effective against the SU 86.86 cancer cell line. It is well-established that cancerous cells can evade apoptosis, which is a characteristic of cancer (45). Our results confirmed that EGCG has anti-tumor potential, inhibits human pancreatic cells' growth, and ultimately induces apoptosis. The cell lines MIAPaCa-2 and SU 86.86 were treated with increasing concentrations of EGCG to determine whether the inhibition of NF-κB by EGCG affects cell apoptosis. The results showed a significant increase in the percentage of apoptotic cells with increasing concentrations of EGCG.

The NF-κB is constitutively overexpressed in various cancer, including breast, lung, liver, pancreatic, prostate, and many types of lymphoma (10). Our covalent docking studies showed EGCG as a covalent inhibitor bound to NF-κB stably and thus, act as a strong inhibitor of NF-κB. The docking studies were further complemented by *in vitro* experiments which showed that EGCG binds to the p65 subunit of NF-κB and triggers apoptosis in pancreatic cell lines. These findings imply that EGCG binds effectively to the NF-κB that may serve as its potential inhibitor and can be further explored.

## 4. Conclusion

The results from covalent docking suggest that the catechin derivatives, EC, ECG, EGC, and EGCG react with sulfhydryl S-atom of Cys-38 of NF-κB p65 subunit and form Cys adducts that hang on the surface of the protein and thus, act as covalent inhibitors of NF-κB. The study proposes the catechin derivatives as potential covalent inhibitors of NF-κB and delineates the detailed binding conformation and molecular interactions. Interestingly, the EC and its galloyl derivative, ECG, had similar binding conformations and the same set of interacting residues. Similarly, the EGC and its galloyl derivative, EGCG were found to have comparable binding conformations. Our experimental results suggest that EGCG reduced cell growth, proliferation, and induced apoptosis in pancreatic cancer cell lines and inhibited NF-κB activity. Furthermore, it repressed the expression of various target genes of NF-κB, such as MMP9, MMP2, cMyc, and BCL-2. Thus, our experimental findings further reinforce the possible usage of EGCG against pancreatic cancer associated with the abnormal activity of the NF-κB.

## Data availability statement

The raw data supporting the conclusions of this article will be made available by the authors, without undue reservation.

## Author contributions

Conceptualization, investigation, writing—original draft preparation, supervision, project administration, and funding acquisition: MS. Methodology: MT. Software and docking and visualization: MR. Validation: MS and MR. Formal analysis: ST and AH. Resources: TZ. Data curation: AH and ST. Writing—review and editing: MT, MR, AH, ST, and TZ. All authors contributed to the article and approved the submitted version.
